# Leporid immunoglobulin G shows evidence of strong selective pressure on the hinge and CH3 domains

**DOI:** 10.1098/rsob.140088

**Published:** 2014-09-03

**Authors:** Ana Pinheiro, Jenny M. Woof, Tereza Almeida, Joana Abrantes, Paulo C. Alves, Christian Gortázar, Pedro J. Esteves

**Affiliations:** 1 CIBIO Centro de Investigação em Biodiversidade e Recursos Genéticos, InBio Laboratório Associado, Universidade do Porto, Campus Agrário de Vairão, Vairão 4485-661, Portugal; 2 Departamento de Biologia, Faculdade de Ciências, Universidade do Porto, Porto 4169-007, Portugal; 3 SaBio IREC (CSIC-UCLM-JCCM), Ronda de Toledo s/n, Ciudad Real 13071, Spain; 4 Division of Cancer Research, Medical Research Institute, University of Dundee Medical School, Ninewells Hospital, Dundee DD1 9SY, UK; 5 Wildlife Biology Program, College of Forestry and Conservation, University of Montana, Missoula, MT 59812, USA; 6 CESPU, Instituto de Investigação e Formação Avançada em Ciências e Tecnologias da Saúde, Gandra PRD, Portugal

**Keywords:** rabbit, *IGHG*, genetic diversity, immunoglobulins constant region, hinge region, evolution

## Abstract

Immunoglobulin G (IgG) is the predominant serum immunoglobulin and has the longest serum half-life of all the antibody classes. The European rabbit IgG has been of significant importance in immunological research, and is therefore well characterized. However, the IgG of other leporids has been disregarded. To evaluate the evolution of this gene in leporids, we sequenced the complete *IGHG* for six other genera: *Bunolagus*, *Brachylagus*, *Lepus*, *Pentalagus*, *Romerolagus* and *Sylvilagus*. The newly sequenced leporid *IGHG* gene has an organization and structure similar to that of the European rabbit IgG. A gradient in leporid IgG constant domain diversity was observed, with the CH1 being the most conserved and the CH3 the most variable domain. Positive selection was found to be acting on all constant domains, but with a greater incidence in the CH3 domain, where a cluster of three positively selected sites was identified. In the hinge region, only three polymorphic positions were observed. The same hinge length was observed for all leporids. Unlike the variation observed for the European rabbit, all 11 *Lepus* species studied share exactly the same hinge motif, suggesting its maintenance as a result of an advantageous structure or conformation.

## 2. Introduction

Immunoglobulin G (IgG) is the predominant serum immunoglobulin. IgG participates directly and indirectly to immune responses through its effector functions, elicited through neutralization and binding to Fcγ receptors (FcγRs) and C1q. These functions include antibody-dependent cellular cytotoxicity, antibody-dependent cell phagocytosis and/or complement-dependent cytotoxicity [1]. IgG also participates in direct neutralization of toxins and viruses. Furthermore, IgG is the only Ig class that binds to FcRn, the neonatal Fc receptor, which provides passive immunity to newborns as well as protecting this particular Ig class from degradation, granting IgG the longest serum half-life of all Ig classes [2]. In mammals, the number of IgG subclasses ranges from 1 to 7, each subclass differing in effector functions [3]. Four IgG subclasses have been found in humans [4], mice [5] and rats [6], three in cattle [7], six in pigs [8] and seven in horses [9]. In bats, the number of IgG subclasses ranges from 1 to 5 depending on the particular species [10]. Among mammals, the European rabbit is seemingly unique in that it possesses only one IgG subclass [11].

**Figure 1.**
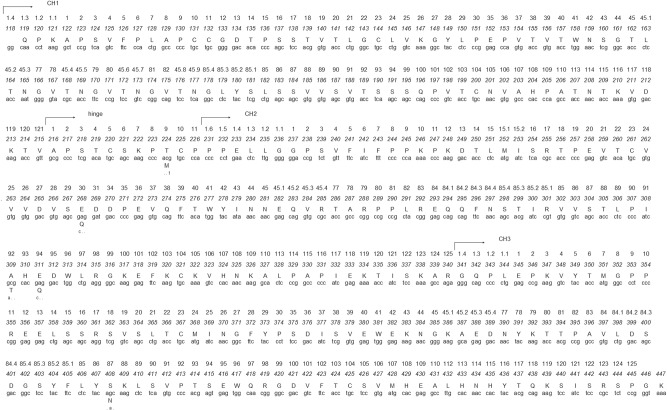
European rabbit (*O. cuniculus*) IgG DNA sequence of constant and hinge regions (GenBank accession number L29172). The amino acid translation is shown above the nucleotide sequence; for polymorphic positions, other amino acid possibilities are shown below the nucleotide sequence. The IMGT unique numbering for the constant domain [13] and EU numbering (in italic type) are shown on top.

The European rabbit IgG has been extensively studied and its allelic variation characterized in detail. Two loci were distinguished by serology, the *d* and *e*, each with two segregating alleles, *d11*/*d12* and *e14*/*e15*, respectively [12]. The *locus d* is correlated to a Thr–Met change at position 9 (IMGT numbering [13]) in the *IGHG* hinge region. As for *locus e*, it is correlated to an Ala–Thr change at position 92 in the *IGHG* CH2 (IMGT unique numbering for C-DOMAIN [13]). Serologic studies have found the *e15* allotype in species of the genera *Oryctolagus*, *Lepus*, *Sylvilagus*, *Romerolagus* and *Ochotona* [14,15], but have failed to identify the *d11* and *d12* allotypes in *Lepus* and *Sylvilagus* genera [16]. Protein and nucleotide sequence data for *IGHG* in Leporids are scarce. The relationship between serology and protein variation at the *e locus* or *IGHG* CH2 domain has been studied by amino acid sequencing of tryptic peptides in various lagomorph species [14,17–20]. The nucleotide sequencing data are limited essentially to the *IGHG* molecule sequence for the European rabbit, and *IGHG* hinge and *IGHG* CH2 domains for a restricted number of *Sylvilagus* and *Lepus* species. Sequencing of the *IGHG* hinge domain in Leporids showed differences between species at residues 8 and 9 (IMGT numbering [13]) [21], whereas in the *IGHG* CH2 a hotspot of variation was found at position 92 (IMGT numbering) [22] (see figure 1).

The family Leporidae comprises 11 genera: *Brachylagus*, *Bunolagus*, *Caprolagus*, *Lepus*, *Nesolagus*, *Oryctolagus*, *Pentalagus*, *Poelagus*, *Pronolagus*, *Romerolagus* and *Sylvilagus*) [23,24]. *Lepus* is a polytypic, cosmopolitan genus, comprising 24–30 currently recognized species [24–26], and most probably originated in North America [27,28]. The remaining genera have more restricted distributions, though having a nearly worldwide distribution. Most, with the exception of *Nesolagus*, *Pronoloagus* and *Sylvilagus*, which comprise two, four and more than 16 species, respectively, are monotypic genera [29–34]. Some discrepancies exist between molecular and fossil data, confusing leporid taxonomy [27,28]. Molecular analyses based on nuclear and mitochondrial markers suggest that within Leporidae two groups diverged around 14 Ma: a first group encompasses the genera *Nesolagus*, *Poelagus* and *Pronolagus*, and the second group includes the remaining eight genera. Within this second group, *Romerolagus* was the first genus to diverge, around 13 Ma, followed by *Lepus*, which diverged around 12 Ma. *Brachylagus* and *Sylvilagus* diverged from a group composed of *Bunolagus*, *Caprolagus*, *Oryctolagus* and *Pentalagus* around 10 Ma. The *Brachylagus*–*Sylvilagus* and *Bunolagus–Caprolagus–Oryctolagus*–*Pentalagus* splits are estimated at 9 Ma (approximate times [28]).

In this study, we extend the knowledge on this immunoglobulin class in leporids by sequencing the complete *IGHG* gene for six additional extant leporid genera: *Bunolagus*, *Brachylagus*, *Lepus*, *Pentalagus*, *Romerolagus* and *Sylvilagus*.

## 3. Material and methods

Total genomic DNA specimens of *Bunolagus*, *Brachylagus*, *Lepus*, *Pentalagus*, *Romerolagus* and *Sylvilagus* genera were extracted from frozen liver or ear tissue using an EasySpin Genomic DNA Minipreps Tissue Kit (Citomed). Additionally, six European rabbits were also analysed: two individuals of the subspecies *Oryctolagus cuniculus algirus*, three individuals of the subspecies *Oryctolagus cuniculus cuniculus* and a domestic rabbit belonging to the New Zealand White breed. PCR amplification of the four *IGHG* exons was conducted using primers designed on the basis of European rabbit *IGHG* available sequences (GenBank accession number AY386696 [35]). A fragment containing the IGHG CH1 and hinge domains was amplified using primers FG12 (5′ TCAGGCCCAGACTGTAGACC 3′) and RE [21] under the following conditions: 15 min at 95°C followed by 35 cycles at 94°C (30 s), 63°C (30 s) and 72°C (45 s), with a final extension at 60°C (20 min). Another fragment containing IGHG CH2 and CH3 exons was amplified using primers F3 [22] and RG31 5′ TTGGAAGGAATCAGGACAGC 3′ under the following conditions: 15 min at 95°C followed by 35 cycles at 94°C (30 s), 60°C (30 s) and 72°C (45 s), with a final extension at 60°C (20 min). Primers were designed so the two fragments overlap in order to obtain the full intron sequence. Sequences were determined by automated sequencing following the Big Dye Terminator Cycle Sequencing protocol (Perkin Elmer, Warrington, UK) using the referred primers.

To confirm the hinge length a fragment of the expressed IgG was also sequenced for one *Lepus granatensis*, one *Lepus europaeus* and one *Sylvilagus floridanus*. Total RNA was extracted from spleen samples using RNeasy Mini Kit (Qiagen, Hilden, Germany), following first strand cDNA synthesis using oligo(dT) as primer (Invitrogen, Carlsbad, CA, USA) and SuperScript III reverse transcription (Invitrogen) as recommended by the manufacturer. A mid-CH1 to mid-CH2 fragment was PCR amplified using primers FG1int2 (5′ CCAGTGACCGTGACTTGGAA 3′) and RG2int2 (5′ GGACTTTGCACTTGAACTCC 3′) designed in conserved regions of the leporid *IGHG* gene segment. A touchdown PCR was performed and the conditions were as follows: 3 min at 98°C followed by five cycles at 98°C (30 s), annealing starting at 66°C with a 1°C decrease/cycle until reaching 62°C (30 s) and 72°C (30 s), followed by 30 cycles at 98°C (30 s), 62°C (30 s) and 72°C (30 s), with a final extension at 72°C (5 min).

Sequences obtained in this study were edited and aligned using CLUSTAL W [36] as implemented in BioEdit software [37] and the amino acid sequences were inferred using BioEdit [37]. The obtained sequences were also aligned and compared with leporid sequences available in GenBank. Accession numbers for all sequences are given in table 1. Codon numbering is according to the IMGT unique numbering for C-DOMAIN [13]. Amino acid residue numbering is also defined according to Eu numbering [38]. Sequence nucleotide diversity was estimated using DnaSP v. 5.10 [39].

**Table 1.** *IGHG* sequences accession numbers for sequences used in this study.

sequence identification	accession number
* rabbit germline sequences*	AY386696; L29172
* rabbit cDNA sequences*	DQ402474, M16426, K00752, XM_002723875, J00665
* Lepus capensis germline CH2*	AJ295218
* Lepus californicus germline CH2*	AJ295219
* Lepus americanus germline CH2*	AJ431716
* Lepus saxatilis germline CH2*	AJ295222
* Lepus timidus germline CH2*	AJ295216
* Sylvilagus cunicularis germline CH2*	AJ295223
* Sylvilagus floridanus germline hinge*	DQ206979
this study	
* *germline	
* Oryctolagus cuniculus cuniculus*	KJ807306–KJ807308
* Oryctolagus cuniculus algirus*	KJ807309–KJ807310
* Oryctolagus cuniculus* New Zealand White breed	KJ807311
* Lepus californicus*	KJ807312, KJ807313
* Lepus callotis*	KJ807314, KJ807315
* Lepus castroviejoi*	KJ807316
* Lepus europaeus*	KJ807317–KJ807324
* Lepus granatensis*	KJ807325–KJ807331
* Lepus saxatilis*	KJ807332, KJ807333
* Lepus townsendi*	KJ807334, KJ807335
* Lepus americanus*	KJ807336, KJ807337
* Lepus corsicanus*	KJ807338, KJ807340
* Lepus capensis*	KJ807341, KJ807343
* Lepus timidus*	KJ807344, KJ807345
* Pentalagus furnessii*	KJ807346
* Sylvilagus floridanus*	KJ807347
* Sylvilagus bachmanii*	KJ807348
* Bunolagus monticularis*	KJ807351
* Brachylagus idahoensis*	KJ807350
* Romerolagus diazii*	KJ807349
* *cDNA	
* Lepus europaeus*	KJ807353
* Lepus granatensis*	KJ807352
* Sylvilagus floridanus*	KJ807354

The secondary structure of the leporids IgG heavy chain was analysed using the DiAminoacid Neural Network Application (DiANNA) (http://clavius.bc.edu/~clotelab/DiANNA/) [40–42]. DiANNA predicts the disulfide connectivity using a neural network trained on databases derived from high-quality protein structures that include evolutionary and secondary structure information. First PSIPRED is run to predict the secondary structure, and this information is then used to find pairs of cysteines using a maximum weight matching.

The nucleotide sequences' alignment was screened for recombination as it can mislead positive selection analysis [43,44]. For this, the software GARD (Genetic Algorithm for Recombination Detection) [45,46], available from the DATAMONKEY web server, was used. The best-fitting nucleotide substitution model was determined using the automatic model selection tool available on the server.

To identify signatures of selection on leporid IgG, we compared the rate per site of non-synonymous substitution (dN) with the rate per site of synonymous substitutions (dS) in a maximum-likelihood (ML) framework, using six different methods. As each of the methods employs unique algorithms, and as done previously [47–49], we only considered those codons identified by at least two of the ML methods to be positively selected codons (PSCs). Using the CODEML program of the PAML v. 4.4 package [50,51], we compared two disparate models—M8, which allows for codons to evolve under positive selection (dN/dS > 1) and M7, which does not (dN/dS ≤ 1)—using a likelihood ratio test with 2 d.f. [52,53]. Codons under positive selection for model M8 were identified using a Bayes Empirical Bayes approach [54] and considering a posterior probability of more than 90%. Using Mega 5 [55], a neighbour-joining phylogenetic tree was used as a working topology, with the p-distance substitution model and the pairwise deletion option to handle gaps and missing data. The obtained tree was in accordance with the accepted lagomorph phylogeny. The five methods for detecting positive selection available from the DATAMONKEY web server [56] were also used: the Single Likelihood Ancestor Counting (SLAC) model, the Fixed Effect Likelihood (FEL) model, the Random Effect Likelihood (REL) model, the Mixed Effects Model of Evolution (MEME) and the Fast Unbiased Bayesian Approximation (FUBAR). For these analyses, the best-fitting nucleotide substitution model was first determined through the automatic model selection tool available on the server.

The location within the IgG structure of the residues under positive selection was analysed by mapping the residues onto the solved crystal structures of rabbit IgG–Fab (PDB ID: 4HBC [57]) and Fc (PDB ID: 2VUO [58]). To examine their relation to putative sites of interest, the sites of interaction with FcγRs, FcRn and complement C1q [2,59–61] were also mapped onto the three-dimansional IgG–Fc structure. The NCBI application Cn3D v. 4.1 (www.ncbi.nlm.nih.gov/Structure/CN3D/cn3d.shtml [62]) was used to this purpose.

## 4. Results

The *IGHG* gene newly sequenced for six leporid genera, *Bunolagus*, *Brachylagus*, *Lepus*, *Pentalagus*, *Romerolagus* and *Sylvilagus*, is similar to the European rabbit (*Oryctolagus*) *IGHG*, and thus the intron–exon organization was inferred from available published rabbit *IGHG* genes. Splicing signals were present at the intron boundaries and hence it is assumed that all studied leporids share the same *IGHG* exon organization as the European rabbit.

### 4.1. CH1 domain

**Figure 2.**
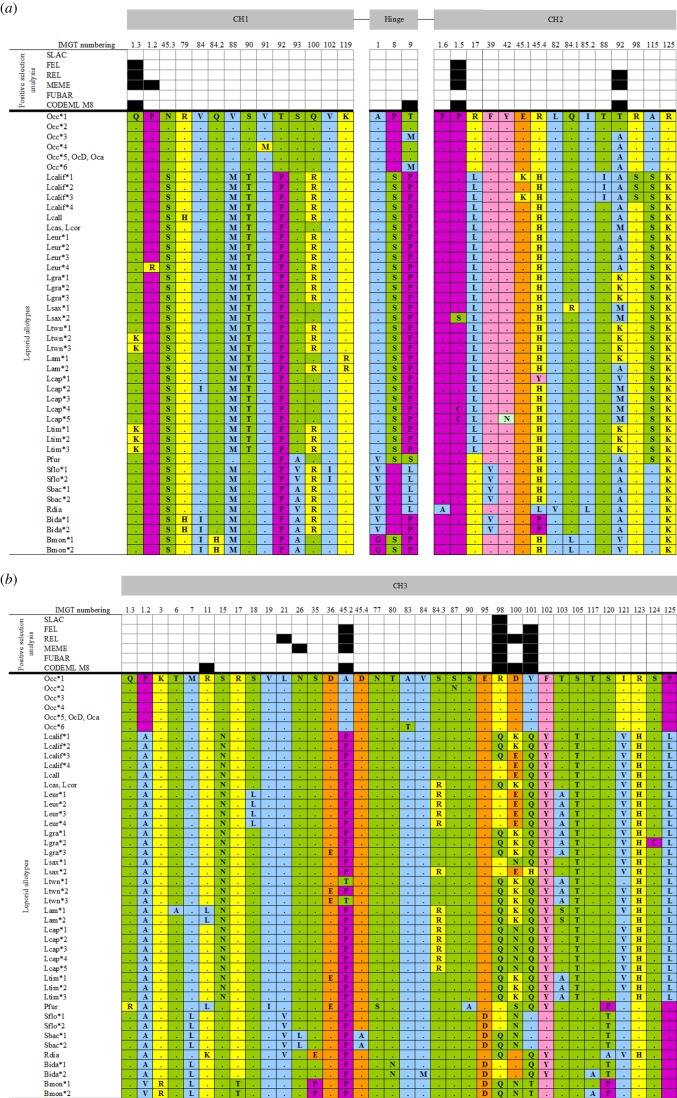
Variable codons and amino acid physico-chemical properties for leporid *IGHG* (*a*) CH1 and CH2 domains and hinge region, and (*b*) CH3 domain. The codons identified as under positive selection by each one of the six methods used are indicated; only those codons identified by at least two methods are considered to be PSCs. The codon numbering is according to the IMGT unique numbering for the constant domain [13]. The colours represent amino acid properties: polar neutral (green), polar positive (yellow), polar negative (orange), non-polar neutral (purple), non-polar aliphatic (blue) and non-polar aromatic (light pink). Occ, *Oryctolagus cuniculus cuniculus*; OcD, domestic rabbit New Zealand White breed; Oca, *Oryctolagus cuniculus algirus*; Lcalif, *Lepus californicus*; Lcall, *Lepus callotis*; Lcas, *Lepus castroviejoi*; Lcor, *Lepus corsicanus*; Leur, *L. europaeus*; Lgra, *L. granatensis*; Lsax, *Lepus saxatilis*; Ltwn, *Lepus townsendi*; Lam, *Lepus americanus*; Lcap, *Lepus capensis*; Ltim, *Lepus timidus*; Pfur, *Pentalagus furnessii*; Sflo, *S. floridanus*; Sbac, *Sylvilagus bachmanii*; Rdia, *Romerolagus diazii*; Bida, *Brachylagus idahoensis*; Bmon, *Bunolagus monticularis.*

The CH1 domain is the most conserved among leporid IGHG domains with only 14 amino acid variable positions, the majority of which involve one substitution, and half are conservative regarding the amino acid properties. The European rabbit IgG differs from the IgG of other leporids by the Asn/Ser change at position 45.3 and by the Thr/Pro change at position 92. *Bunolagus* IgG has a His for a Glu substitution at position 84.2 and *Lepus* IgG has a Thr for a Ser change at position 90. Only the substitution at position 84.2 involves changes in amino acid characteristics (figure 2*a*).

### 4.2. CH2 domain

The CH2 domain of leporid IgGs, though fairly conserved, shows more diversity than the CH1 domain. For the CH2 domain, 15 amino acid variable positions were observed, 11 of which involve one substitution but, contrary to that observed for the CH1 domain, the majority of these polymorphic positions involve changes in amino acid properties. The European rabbit has specific Arg residues at positions 45.4 and 125. The residues Leu17, Pro45.4 and Leu84.1 are specific to *Lepus*, *Brachylagus* and *Bunolagus*, respectively. *Romerolagus* uniquely has Ala1.6, Leu45.4, Val82 and Leu85.2 residues (figure 2*a*).

### 4.3. CH3 domain

This is the most diverse of the leporid *IGHG* domains with 36 variable positions, the majority involving changes in amino acid properties. It also has the highest number of diagnostic positions. In this domain, the European rabbit has specific Pro1.2 and Ala45.2 residues, *Bunolagus* uniquely has Val1.2, Arg3, Thr17 and Pro35 residues, and *Brachylagus* has an Asn for a Thr change at position 80. Three diagnostic positions were observed for each of three genera: *Lepus* at Asn15, Thr105 and Leu125 residues, *Pentalagus* at Arg1.3, Ser77 and Ala90, and *Romerolagus* at Lys11, Glu35 and Ala120 as distinctive. Only *Sylvilagus* lacked specific residues (figure 2*b*).

### 4.4. Hinge

Three variable positions were found, all involving changes in amino acid properties. Changes at these positions define genera-specific motifs, with the exception of *Sylvilagus* and *Romerolagus*, which share the same substitutions: Val1, Pro8 and Leu9 (figure 2*a*). For all studied leporids, an 11-residue hinge was observed, which, given the high variability observed in IgG hinge length in other mammals, is surprising. To check whether alternative splicing sites could be used by some leporids, the expressed hinge was sequenced for *Lepus* and *Sylvilagus* individuals, and each one had the same 11 residues.

### 4.5. Intron diversity

The intronic regions between CH1–hinge, hinge–CH2 and CH2–CH3 were fully sequenced. Overall nucleotide diversity for these regions is similar to that observed for the coding regions (Pi_introns_ = 0.03704; Pi_exons_ = 0.03277). The major differences observed between leporid genera are insertions and deletions (indels), which reflect the accepted leporid phylogeny. Indeed, in the intron between CH1 and hinge exons, a 20 bp deletion is shared by *Bunolagus*, *Oryctolagus* and *Pentalagus*, whereas *Romerolagus* has a unique 10 bp deletion. Similarly, in the intron between CH2 and CH3 exons, a 1 bp deletion is shared by *Brachylagus* and *Sylvilagus*, whereas *Oryctolagus* has two characteristic 1 bp deletions and *Romerolagus* has an insertion of 13 bp (electronic supplementary material).

### 4.6. Leporid IgG heavy chain structure

All immunoglobulin heavy chains are organized into globular domains, a structure stabilized by intra-chain disulfide bonds between conserved cysteines in each domain at positions 23 and 104 in each domain. As expected, all studied leporids share these cysteines. The European rabbit IgG further has two additional cysteines in the CH1 domain at positions 10 and 11 (Cys131 and Cys132; Eu numbering) and a further two in the hinge, at positions 5 and 10. The CH1 domain Cys at position 10 (Cys131; Eu numbering) bonds to the light chain Cys, the CH1 Cys at position 11 (Cys132; Eu numbering) bonds to the Cys at position 5 in the hinge, and the other hinge Cys forms the inter-chain disulfide bridge with its equivalent in the other heavy chain. Again, conserved cysteines at these positions are shared by all studied leporids, suggesting that the IgG heavy chain structure is maintained across leporids. However, extra cysteines were found in the CH2 domain at position 1.5 (Cys232; Eu numbering) of two *Lepus capensis* alleles and in the CH3 domain at position 124 (Cys444; Eu numbering) for one *L. granatensis* allele. Disulfide bond prediction analysis indicates that the extra CH2 cysteine does not establish any bond. However, for the allele with the additional CH3 Cys, different bonds to those described above are predicted for the CH2 and CH3 domains. The CH2 domain Cys at position 23 (Cys261; Eu numbering) is now predicted to bond with the CH3 domain Cys at position 104 (Cys425; Eu numbering), and the CH3 domain Cys at position 23 (Cys367; Eu numbering) is predicted to bond with the CH3 domain Cys at position 124 (Cys444; Eu numbering). However, the physiological relevance of these predictions remains unclear.

Glycosylation is also important for protein structure and function. The European rabbit IgG Fc has an N-glycosylation site at CH2 84.4 (Asn297; Eu numbering), which was found to be conserved in all studied leporids, and indeed all vertebrate IgG. No other N-glycosylation sites were predicted. O-linked glycans (i.e. glycans linked to Ser/Thr residues in Ser/Thr/Pro-rich domains are known on human IgA1 and IgD hinges) and also on the rabbit IgG hinge, for which the Thr residue at hinge position 9 of d12 rabbits is O-glycosylated (d11 rabbits hinge position 9 have a Met residue) [16,63,64]. This O-glycan confers protection against cleavage of the rabbit IgG hinge [63]. Putative O-glycosylation sites are present on *Lepus* and *Bunolagus* hinge position 8, and *Pentalagus* hinge positions 8 and 9, all occupied by Ser residues.

### 4.7. Signatures of positive selection on leporid immunoglobulin G

**Figure 3.**
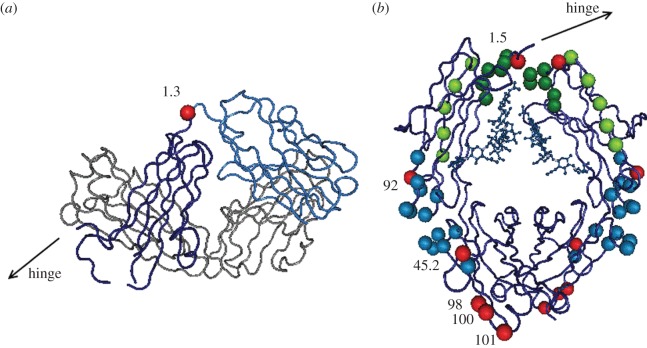
Leporid residues encoded by PSCs in the three-dimensional structure of rabbit IgG. (*a*) Rabbit IgG–Fab (PDB ID 4HBC). (*b*) Rabbit IgG–Fc (PDB ID 2VUO). The light chain is in the background coloured grey, the heavy chain variable domain is in the foreground coloured light blue and the heavy chain constant domains are coloured dark blue. Positively selected codons are represented in red dots. Residues significant for FcγR interaction are highlighted in dark green, residues significant for FcRn interaction are in light blue and residues significant for C1q complement interaction are in light green. Residue numbering is according to IMGT unique numbering for the constant domain [13]. The N-glycan attached to CH2 84.4 (Asn297; Eu numbering) is shown in ball and stick representation.

The comparison of the two disparate models implemented in CODEML revealed evidence for positive selection acting on the leporid IgG, with the model allowing sites to evolve under positive selection (M8) showing a significantly better fit than the model that did not (M7) (lnL M7/lnL M8 =−2850.1/−2817.1; −2ΔlnL = 33; *α* < 0.01). Comparing the sites recognized by each of the six employed methods led to the identification of seven PSCs. One locates to the CH1 domain, two to the CH2 domain and four to the CH3 domain. All of these residues show changes in amino acid characteristics and occupy exposed positions on the European rabbit IgG structure. Interestingly, four of these codons locate near sites of interaction with ligands: the CH1 residue 1.3 (residue 119; Eu numbering) is in the immediate vicinity of the VH domain, the CH2 residue 1.5 (residue 232; Eu numbering) lies in the region of residues that interact with FcγRs, and the CH2 92 residue (residue 309; Eu numbering) and CH3 45.2 residue (residue 387; Eu numbering) locate on the region of residues that interact with FcRn. Of note, the CH3 domain residues at positions 98, 100 and 101 (residues 419, 421 and 422; Eu numbering) form an exposed cluster in the C-terminal portion of this domain (figure 3).

## 5. Discussion

Rabbit IgG has been extensively studied and its genetic diversity thoroughly characterized, but, despite the relevance of IgG as a crucial component of the host immune response and the uniqueness of rabbit IgG, the extension of this knowledge to other leporids has been neglected. In this work, we extended knowledge on the evolution of leporid IgG by analysing six extant genera.

The results obtained in this study reveal that the leporids share considerable sequence similarity for their *IGHG* (approx. 94%). A gradient in constant domain diversity is observed with the CH1 domain being the most conserved of leporid *IGHG* domains and the CH3 domain the most variable. In fact, there are twice as many variable amino acid sites and number of diagnostic substitutions in the CH3 domain compared with the CH1 and CH2 domains, making the CH3 the most informative domain for leporid species identification. The CH3 domain has been identified as the most diagnostic domain to distinguish between IgG isotypes for swine [8] and primate species [65]. The divergence previously found for IgG CH3 domains between macaques and human species that diverged around 32 Ma [66] is similar to the divergence found between *Oryctolagus* and *Romerolagus*, genera that separated around 13 Ma [28], and thus it seems that either the leporid IgG CH3 domain is evolving under selective pressure to change or that evolutionary constraints are conserving primate IgG CH3 domains.

Despite the overall conservation found for leporid *IGHG*, hotspots of variability exist in the hinge and CH2 and CH3 domains, and we found evidence of positive selection acting on all IgG constant domains. Previous studies of leporid *IGHG* described the hinge position 9 and CH2 position 92 as hotspots of variability [21,22]. Our results, including more genera and species than former studies, confirm that hinge position 9 is a leporid hotspot of variability, while the CH2 position 92 residue (residue 309; Eu numbering) proves to be a *Lepus*-specific hotspot, with four different residues in this genus but only 2 in other leporids. Additionally, we can pinpoint as having high amino acid diversity the CH2 position 45.4 (residue 387; Eu numbering) and CH3 position 100 (residue 421; Eu numbering), each having five different residues in the studied leporids. These hotspots of variability, and also the seven positions identified as positively selected, exhibit changes in amino acid physico-chemical properties, which may impact on the IgG conformation and structure. Changes at the positively selected CH1 1.3 residue (residue 119; Eu numbering) may impact on the antigen-binding site conformation. This would possibly improve leporid antigen-binding possibilities given that the usage of the VH1 gene in 90% of VDJ rearrangements in leporids confers a somewhat restricted diversity to the leporid VH domain [67–70]. On the other hand, changes at PSCs CH2 1.5 (residue 232; Eu numbering), CH2 92 residue (residue 309; Eu numbering) and CH3 45.2 residue (residue 387; EU numbering) may influence binding of IgG to Fc receptors. In particular, the positively selected CH2 position 1.5 (residue 232; Eu numbering) lies in close vicinity to CH2 residues 1.3–1 (234–237; Eu numbering), which in human IgG form the core of the interaction site for FcγR [60,71]. As IgG from the European rabbit binds human FcγRI with affinity comparable with human IgGs [72], consistent with a similar interaction mode across species, one might speculate that variation at this position may prove adaptive by influencing binding of IgG to rabbit receptors. The changes at PSCs CH2 1.5, CH2 92 and CH3 45.2 may also confer some resistance against proteins produced by some bacterial pathogens that target the lower hinge–proximal CH2 region (e.g. IdeS [73]) and the CH2–CH3 interface (e.g. Staphylococcal protein A and Streptococcal protein G [74]). Interestingly, it has been noted that bacterial pathogens in different mammalian species also target this same inter-domain region in immunoglobulins [75], so it is possible that bacterial species evolved to infect leporids may employ a similar evasion strategy of production of proteins that bind the CH2–CH3 interface.

In contrast to what was found for mammalian IgA, where the Cα3 domain showed less evidence of having evolved under positive selection than Cα1 or Cα2 [48], the leporid IgG CH3 domain has the highest number of positively selected sites of all IgG constant domains, showing that this domain is evolving under positive selection. Areas across the surface of the CH3 domain have been implicated in the formation of hexameric IgG that assembles at antigenic cell surfaces, recruits C1q and activates complement [76]. Thus, the cluster of PSCs observed in this region suggests that the C-terminal CH3 has some functional relevance in the leporids and could be related to protective complement-mediated mechanisms against specific pathogens.

The hinge region shows considerable variability both in amino acid composition and length among IgA and IgG subclasses and alleles, and across species (e.g. [8,10,77]). The hinge is the preferential target region for proteolytic cleavage by numerous bacterial proteases (discussed in [68,78]), which could explain the great variability observed for this antibody region. Given this context, the lack of variation observed in this study for the *Lepus* IgG hinge is particularly interesting. Previous studies by Esteves *et al.* [21] indicated that leporids share the same hinge length and have amino acid differences at only two hinge positions, 8 and 9 (IMGT numbering). The European rabbit shows two residues at position 9, Met and Thr, which correlate with the serological allotypes d11 and d12 [12,79]. The d12 Thr residue is O-glycosylated, conferring protection against cleavage of the rabbit IgG hinge [63]. Thus, the glycosylation of rabbit, *Lepus*, *Bunolagus* and *Pentalagus* hinge may protect the IgG from effects of proteases of pathogens and tumour cells. Despite this increased resistance against proteolytic attack of the d12 allotype, both allotypes interact with FcγR equally well (e.g.[72]). We have confirmed that all seven studied leporid genera have an 11-residue hinge, like *Oryctolagus*, and that this hinge is expressed by *Lepus* and *Sylvilagus*. Thus, one can assume that most likely all leporids use a short 11-residue hinge. The existence of more than one IgG in leporids other than the European rabbit has so far not been assessed. Thus, despite having found no evidence for the existence of more than one *IGHG* copy in the studied leporid genera during the course of this work, we cannot exclude the possibility of additional IgG genes in leporids, although it seems highly unlikely. Unlike the variation observed for the European rabbit, all 11 *Lepus* species studied share exactly the same hinge motif, indicating that it may be maintained due to an advantageous structure or conformation.

## Supplementary Material

Alignment of the leporid IGHG nucleotide sequences
